# Female infertility and dietary antioxidant index (DAI); a case-control study

**DOI:** 10.1186/s12905-023-02747-9

**Published:** 2023-11-16

**Authors:** Roya Kabodmehri, Fatemeh Sadat Hashemi Javaheri, Farkhondeh Alami, Zahra Mahmoudi, Arezoo Amjadi, Zahra Saeedirad, Saeed Omidi, Shiva Sadeghi, Mohadeseh Sadat Mousavi Hoseini, Zahra Mohamadiyan, Zahra Salimi, Hanieh Shafaei, Reyhaneh Rasekhmagham, Seyedeh Hajar Sharami, Maryam Karimian, Hoora Karimi, Saeid Doaei

**Affiliations:** 1 Reproductive Health Research Center, Department of Obstetrics and Gynecology, School of Medicine, Al-Zahra Hospital, Guilan University of Medical Sciences, Rasht, Iran; 2 Department of Clinical Nutrition, School of Nutrition and Food Sciences, Tabriz University of Medical Sciences, Tabriz, Iran; 3 Student Research Committee, Department of Nutrition, Faculty of Medicine, Urmia University of Medical Sciences, Urmia, Iran; 4 Department of Nutrition, Science and Research Branch, Islamic Azad University, Tehran, Iran; 5 Department of Nutrition, School of Nutritional Sciences and Food Technology, Kermanshah University of Medical Sciences, Kermanshah, Iran; 6 Department of Clinical Nutrition and Dietetics, Faculty of Nutrition and Food Technology, Shahid Beheshti University of Medical Sciences, Tehran, Iran; 7 Department of Health Education and Promotion, Research Center of Health and Environment, School of Health, Guilan University of Medical Sciences, Rasht, Iran; 8 Nursing and Midwifery School, Guilan University of Medical Sciences, Rasht, Iran; 9 Department of Food Science and Technology, Science and Research Branch, Islamic Azad University, Tehran, Iran; 10 Department of Nutrition, Faculty of Medical Sciences, Science and Research Branch, Islamic Azad University, Tehran, Iran; 11 Nutrition and Metabolic Diseases Research Center, Ahvaz Jundishapur University of Medical Sciences, Ahvaz, Iran; 12 Nursing and Midwifery School, Guilan University of Medical Sciences, Rasht, Iran; 13 Department of Endocrinology and Metabolism, School of Medicine, Ilam University of medical sciences, Ilam, Iran; 14 Department of Community Nutrition, Faculty of Nutrition and Food Technology, National Nutrition and Food Technology Research Institute, Shahid Beheshti University of Medical Sciences, Tehran, Iran

**Keywords:** Infertility, Dietary intake, Dietary antioxidant index (DAI)

## Abstract

**Background:**

Adequate intake of natural antioxidants may improve female fertility. The aim of this study was to examine the link between female infertility and dietary antioxidant index (DAI).

**Methods:**

This case-control study was conducted on 125 women with recently diagnosis of reduced ovarian reserves (AMH < 1.1) as the case group and 125 women with normal ovarian reserve as the control group in Rasht, Iran. The amount of food intake was assessed using the food frequency questionnaire (FFQ) and the DAI was calculated to estimate the antioxidant capacity of the diet.

**Results:**

Regarding dietary intake, the infertile women had a lower intake of potassium (2789.25 ± 777 vs. 2593.68 ± 443 mg/d, P = 0.02), magnesium (204.12 ± 66 vs. 189.73 ± 34 mg/d, P = 0.03), copper (0.93 ± 0.40 vs. 0.82 ± 0.20 mg/d, P < 0.01), vitamin C (133.99 ± 46 vs. 122.62 ± 24 mg/d, P = 0.02), and fiber (14.53 ± 3 vs. 13.44 ± 2 g/d, P < 0.05), and a higher intake of cholesterol (205.61 ± 58 vs. 227.02 ± 46 mg/d, P < 0.01) than the control group (All P < 0.05). The DAI was negatively associated with infertility (OR: 0.94, CI 95%: 0.88–0.97, P = 0.03). The association remained significant after adjustments for age, BMI, the underlying diseases, fertility frequency, IVF failure, and calorie intake.

**Conclusion:**

Following an antioxidant-rich diet may reduce the risk of infertility. More longitudinal studies are warranted to confirm these results and discover the underlying mechanisms.

**Supplementary Information:**

The online version contains supplementary material available at 10.1186/s12905-023-02747-9.

## Introduction

The inability to conceive after a year of consistent unprotected sexual activity is considered as the definition of infertility [1]. Approximately 70 million individuals worldwide are influenced by infertility [2]. According to the World Health Organization (WHO), fertility problems affect 9% of couples around the world [2]. The evidence shows that about 8% of Iranian women suffer from lifetime primary infertility [3]. Reduced fertility can be the result of genital infection, endocrine disorders like polycystic ovary syndrome, genetic disorders, immunological factors [4], diabetes, thyroid disorders, maternal age, weight [5], and dietary intake [6, 7]. Recent studies indicated that dietary antioxidants may play a role in fertility and birth outcome [8] and reported the potential connection between oxidative stress (OS) and low antioxidant levels with infertility. The occurrence of oxidative stress is resulted from decreased antioxidant consumption and increased antioxidant utilization due to excessive production of reactive oxygen species (ROS).

The findings of a review study indicated that the intake of recommended amounts of natural antioxidants may decrease OS and enhance female fertility [9]. Some researchers found that the consuming antioxidants such as vitamins C and E may lead to a shorter time to conception in women [10, 11]. Studies have shown that a lower daily intake of magnesium leads to damage to the female and male reproductive system [12]. In rats, retinol supplementation increased the levels of antioxidant enzymes including superoxide dismutase (SOD), catalase (CAT) and glutathione peroxidase (GPX) and also improved fertility conditions [13]. Moreover, a cross-sectional study conducted on infertile men found that selenium and β-carotene intake were linked to higher sperm motility and higher semen volume. However, there was no correlation between zinc and vitamin E intake and semen parameters [14]. Dietary antioxidant index (DAI) has been introduced as a valid indicator for measuring overall antioxidant content in the diet (such as vitamin C, E, A, and Zinc, selenium, and magnesium) [15]. Various studies reported that DAI is associated with chronic diseases such as gastric cancer, colorectal cancer, nonalcoholic fatty liver, and obesity [16].

Several studies between antioxidant status and male infertility have been done [17–19]. However, there are few studies on the effect of dietary antioxidants on female infertility, and a firm association between infertility in women and DAI has not been yet established. So, the present study aimed to investigate the association between female infertility and DAI.

## Methods

This case-control study was conducted in 2022–2023 on infertile patients referred to the infertility clinic of Al-Zahra Hospital, Rasht, Iran. By using the consecutive sampling method, people referred to the infertility center who met the inclusion criteria were included in the study from the baseline until the required sample size was completed. After obtaining informed consent, 125 women with recently diagnosis of reduced ovarian reserves (AMH < 1.1) without previous therapies for ovarian stimulation were recruited as the cases, and 125 women categorically matched for age and body mass index (BMI) but with normal ovarian reserve and with problems in male fertility factor were included as the controls. The sample size was calculated as 246 individuals using Open EPI [23] according to the odds ratio obtained in a previous similar study [24].

Inclusion criteria for the case group were women aged 18 to 40 years with primary or secondary infertility, who consented to participate in the study, and were clinically diagnosed by a gynecologist no more than 3 months ago. Exclusion criteria were included use of drugs affecting the absorption of dietary antioxidants, the use of drugs that inhibit digestive enzymes such as Orlistat, the use of probiotics and/or prebiotics, suffering from diseases affecting the absorption of dietary antioxidants including people with malabsorption and other gastrointestinal problems e.g. irritable bowel syndrome, and inflammatory bowel diseases.

The data collection instruments include a general and a pathological questionnaire, as well as a 168-item semi-quantitative food frequency questionnaire (FFQ) that reflects the food items consumed during last 12 months. The general questionnaire included data on age, education level, occupation, height, weight, and BMI. Data on medical history, history of medication use, consumption of nutritional supplements, hormonal status (including AMH-FSH-LH), and BMI was also collected.

The amount of food intake was assessed using the FFQ questionnaire which has confirmed validity and reliability in the Iranian population [20]. The FFQ consists of 168 food items and was completed to assess the dietary intake of antioxidants during the last year. The information collected by FFQ was transferred to the Nutritionist IV software to evaluate the amount of the intake of macronutrients and micronutrients during a day. Then, the Dietary Antioxidant Index (DAI) was calculated using the method proposed by Wright et al. [21] to estimate the antioxidant capacity of the diet. DAI is a simple and functional method to evaluate diet quality based on dietary antioxidants [22]. The components of DAI are dietary antioxidants including vitamins A, E, C, and selenium, magnesium and zinc. To compute the DAI, the mean intake of vitamins A, C, E, and magnesium, selenium, and zinc is subtracted from the intake of each item individually, and the obtained value is divided by the standard deviation of global intake, yielding the standard value for each item. Finally, the standard value of the items (six items) is summed together to obtain the DAI score, as shown below:

$${\text{DAI}}=\sum _{i=1}^{n=6}.\frac{individual\,intake\,-\,global\,mean}{global\,SD}$$

### Statistical analysis

The t-test (for quantitative variables) and the chi-square test (for qualitative variables) were used to compare the socio-demographic status and food intake of the case and control groups. The association between DAI and infertility was evaluated using logistic regression. The effect of background and confounding variables including age (as quantitative), weight, BMI (as quantitative), consumption of antioxidant supplements, underlying diseases, smoking, alcohol consumption, and physical activity were adjusted in different regression models. SPSS version 21 was used for data analysis, and the significance level for all analyses was considered as P < 0.05.

### Ethical considerations

Participants had the option to participate and leave the study whenever they wanted. The demographic, obstetric, and clinical information remained confidential and all documents were kept in a safe place exclusively accessible by the researchers. The ethical considerations of the present study were approved by the ethics committee of Gilan University of Medical Sciences, Rasht, Iran (Code: IR.GUMS.REC.1401.147).

## Results

The general characteristics and the status of the female sex hormones of the participants are presented in Table 1. The case group had significantly lower AMH (0.52 ± 1.79 vs. 2.30 ± 0.76 ng/mL, P < 0.01) and higher mean of FSH (14.46 ± 18.82 vs. 9.06 ± 8.07 mIU/mL, P < 0.01) and LH (8.03 ± 10.03 vs. 6.03 ± 4.32 mIU/mL, P = 0.043) compared to the control group.

**Table 1:** The characteristics of the participants

Mean ± SD			
	Controls (n = 125)	Cases (n = 124)	p-value
Age (years)	32.92 ± 5.41	37.12 ± 3.50	0.07
Infertility Duration	62.52 ± 49.07	49.60 ± 49.59	0.06
Weight (kg)	71.65 ± 16.26	68.13 ± 13.57	0.06
Height (cm)	157.36 ± 23.05	160.80 ± 30.65	0.32
BMI (kg/m^2^)	27.80 ± 12.53	26.26 ± 5.48	0.21
FSH (mIU/mL)	9.06 ± 8.07	14.46 ± 18.82	< 0.01
LH (mIU/mL)	6.03 ± 4.32	8.03 ± 10.03	0.043
AMH (ng/mL)	2.30 ± 0.76	0.52 ± 1.79	< 0.01

Data are means ± SDs.

Table 2 shows the comparison of dietary intake of nutrients between the case and control groups. The case group had higher intake of cholesterol (227.02 ± 46.05 vs. 205.61 ± 58.88 mg/d, P = 0.002) and lower intake of potassium (2593.68 ± 443.62 vs. 2789.25 ± 777.93 mg/d, p = 0.02), magnesium (189.73 ± 34.78 vs. 204.12 ± 66.94 mg/d, p = 0.03), coper (0.82 ± 0.20 vs. 0.93 ± 0.40 mg/d, P < 0.01), vitamin C (122.62 ± 24.48 vs. 133.99 ± 46.44 mg/d, P = 0.02) and fiber (13.44 ± 2.12 vs. 14.53 ± 3.79 g/d, P = 0.005) compared to the control group. The groups did not differ in their intake of other dietary nutrients.

**Table 2:** Dietary nutrients intake among the case and control groups

	Controls (n = 125)	Cases (n = 124)	P
Calorie (kcal/d)	2012.42 ± 466.88	1955.67 ± 301.27	0.26
Protein (g/d)	71.93 ± 16.30	71.73 ± 9.07	0.91
CHO (g/d)	318.37 ± 88.40	309.74 ± 56.12	0.36
Fat (g/d)	59.53 ± 18.56	57.51 ± 10.13	0.29
Cholesterol (mg/d)	205.61 ± 58.88	227.02 ± 46.05	< 0.01
Sodium (mg/d)	3158.10 ± 1038.45	3309.16 ± 720.90	0.18
Potassium (mg/d)	2789.25 ± 777.93	2593.68 ± 443.62	0.02
Iron (mg/d)	16.88 ± 4.40	17.05 ± 3.01	0.72
Calcium (mg/d)	808.53 ± 255.09	794.98 ± 153.54	0.61
Magnesium (mg/d)	204.12 ± 66.94	189.73 ± 34.78	0.03
Phosphorus(mg/d)	887.24 ± 307.36	857.18 ± 158.35	0.33
Zinc (mg/d)	6.49 ± 2.02	6.32 ± 1.11	0.40
Copper (mg/d)	0.93 ± 0.40	0.82 ± 0.20	< 0.01
Manganese (mg/d)	2.56 ± 0.76	2.50 ± 0.54	0.45
Selenium (µg/d)	0.02 ± 0.01	0.02 ± 0.01	0.11
Fluoride (mg/d)	17493.17 ± 6294.50	18521.51 ± 6501.13	0.21
Chromium(µg/d)	0.005 ± 0.004	0.005 ± 0.003	0.90
Molybdenum (µg/d)	31.72 ± 10.48	33.43 ± 7.62	0.14
Vitamin A (µg/d)	886.24 ± 396.07	829.59 ± 284.77	0.19
Vitamin E (mg/d)	2.82 ± 0.67	2.77 ± 0.61	0.49
Vitamin B1 (mg/d)	1.69 ± 0.49	1.72 ± 0.31	0.71
Vitamin B2 (mg/d)	1.19 ± 0.39	1.19 ± 0.22	0.99
VitaminB3 (mg/d)	19.64 ± 5.14	19.64 ± 3.31	0.99
Vitamin B6 (mg/d)	1.08 ± 0.25	1.05 ± 0.14	0.24
Folate (µg/d)	224.10 ± 62.33	215.68 ± 34.37	0.19
Vitamin B12 (µg/d)	2.73 ± 1.08	2.62 ± 0.60	0.37
Pantothenic acid (mg/d)	4.10 ± 1.34	3.94 ± 0.72	0.24
Biotin (µg/d)	16.68 ± 4.89	17.01 ± 3.67	0.56
Vitamin C (mg/d)	133.99 ± 46.44	122.62 ± 24.48	0.02
Vitamin D (µg/d)	1.17 ± 0.80	1.08 ± 0.50	0.32
Vitamin K(µg/d)	126.41 ± 38.86	130.08 ± 21.79	0.36
Fiber (g/d)	14.53 ± 3.79	13.44 ± 2.12	< 0.01

The amounts of DAI among the case and control group are presented in Table 3. The case group had lower DAI of magnesium (-0.13 ± 0.65 vs. 0.13 ± 1.25 mg/d, P = 0.03), vitamin C (-0.15 ± 0.65 vs. 0.15 ± 1.24 mg/d, P = 0.02) and total DAI (-0.57 ± 3.49 vs. 0.65 ± 5.34 mg/d, P = 0.03) than the control group. There are no differences between cases and control group regarding to the DAI of Vitamin E, Vitamin A, Zinc and selenium.

**Table 3:** The amounts of DAI among the case and control groups

	Controls	Cases	P
Zinc	0.054 ± 1.24	-0.05 ± 0.69	0.40
selenium	0.10 ± 1.22	-0.10 ± 0.71	0.11
magnesium	0.13 ± 1.25	-0.13 ± 0.65	0.03
Vitamin C	0.15 ± 1.24	-0.15 ± 0.65	0.02
Vitamin E	0.04 ± 1.05	-0.04 ± 0.95	0.50
Vitamin A	0.08 ± 1.14	-0.08 ± 0.82	0.20
DAI	0.65 ± 5.34	-0.57 ± 3.49	0.03

The association between infertility and DAI is presented in Table 4. An inverse association was observed between the DAI and infertility (OR: 0.94, CI 95%: 0.88–0.97, P = 0.03). The association remained significant after adjustment for age, BMI, the underlying diseases, fertility frequency, and IVF failure (OR: 0.94, CI 95%: 0.89–0.98, P = 0.04) (Model 2), and after additional adjustment for calorie intake (OR: 0.93, CI 95%: 0.86–0.97, P = 0.04) (Model 3).

**Table 4:** Logistic regression of the association between infertility and DAI

Models	OR	CI95%	P
Model 1	0.94	0.88–0.97	0.03
Model 2	0.94	0.89–0.98	0.04
Model 3	0.93	0.86–0.97	0.04

Model 1: Crude, Model 2: Adjusted for age, BMI, underlying diseases disease, fertility frequency, and IVF failure, Model 3: Further adjustments for calorie intake

## Discussion

The aim of the study was to explore the link between female infertility and the DAI in Iranian women. Regarding the intake of dietary components, the infertile women had a lower significant potassium, magnesium, copper, vitamin C, and fiber intake, and a higher significant cholesterol intake than the control group. In addition, the mean of DAI was higher in the control groups. Our study also showed that DAI was negatively associated with infertility. Therefore, women with higher DAI scores (Implying a noteworthy intake of dietary antioxidants) were found to have a lower risk of infertility. Considering that high dietary quality will result in a high intake of micronutrients with antioxidant activity; thereby a high quality diet may protect against female infertility.

The beneficial effects of some dietary components on infertility were frequently reported [23]. Some studies investigated the association of the DAI components including several types of antioxidants and infertility [9, 24, 25]. The DAI takes into account all dietary antioxidants and their combined effects, rather than analyzing each component separately [26]. Given its strong correlation with serum antioxidant levels, the DAI provides a comprehensive evaluation of dietary antioxidants. In line with our results, various studies reported the association of antioxidants with infertility. Elizabeth et al. [10] assessed the association between dietary antioxidant intake among couples treated for infertility and reported that the intake of the antioxidants such as vitamin C, b-carotene, and vitamin E is related to fertility in women. Another study on the effect of a high antioxidant diet in women with endometriosis [27] reported that infertility is accompanied with higher oxidative stress and antioxidant exhaustion mechanisms, and demonstrated that the daily intake of vitamins C, E, and A is lower in women with infertility compared to other women. Jessica et al. [28] examined the association of maternal plasma concentrations of zinc, copper, and selenium, and time to pregnancy and subfertility and reported that lower selenium and zinc serum concentration is inversely associated with fertility, while there was no relationship with copper. In general, some previous studies found a link between antioxidants status and infertility, but there are discrepancies in the methods used to evaluate the antioxidant capacity of the diet. Based on the results of the present study, the DAI may be a useful tool for evaluating total dietary antioxidant capacity [29].

The exact molecular mechanism of the effects of antioxidants on infertility is not clear. Unexplained infertility may be caused by a disruption of the balance between ROS and antioxidant levels [30]. Vitamin C plays a key role in collagen synthesis in the extracellular matrix of the corpus luteum and the production of progestin needed to maintain the endometrium tissue development [31]. Furthermore, vitamin E helps protect the ovarian surface epithelium from oxidative damage [32, 33]. Hence, enhanced ovarian vitamin E levels help the protection of the aging ovary during luteolysis and the reduction of the ability of luteal cell to eliminate OS [34]. The function of estrogen is depended on magnesium, which aids in the binding of FSH (hormone stimulating the ovaries) to ovary receptors. A lack of magnesium is linked to increased smooth muscle cell tone, which can obstruct a healthy fallopian tube [35, 36].

Overall, the present study had several strengths. First, this study is the first to explore the connection between a new indicator for evaluating antioxidant properties of diet and the risk of female infertility. To our knowledge, no other study has employed the DAI to assess the chances of female infertility, making it challenging to compare results but confirming the novelty of this research. Second, using regression models in this matched case-control study and considering different confounders improved the obtained results.

However, this study had some limitations. One of our study’s limitations was probability of over-estimation and under-estimation due to using self-report FFQ for dietary assessment. Moreover, although there are many nutrients and non-nutritive dietary components that may have antioxidant roles, the DAI indicator includes only six essential micronutrients with antioxidant properties.

## Conclusion

According to this study, DAI can be a practical and effective tool in assessing antioxidant status of diet. Receiving antioxidant-rich foods may decrease the risk of infertility. Based on our research, in order to reduce the odds of infertility, the consumption of antioxidant through the diet is recommended. Hence, following a diet rich in antioxidants can be considered as a useful strategy to reduce the risk of infertility. More studies with larger sample size are needed to confirm the results of the present study and to discover the underlying mechanisms. In addition, future intervention studies are required before recommending antioxidant supplements against infertility.

### Electronic supplementary material

Below is the link to the electronic supplementary material.

Supplementary Material 1

## Data Availability

The datasets used and analyzed during the current study available from the corresponding author on reasonable request.

## References

[1] Carson SA, Kallen AN (2021). Diagnosis and management of infertility: a review. JAMA.

[2] Fainberg J, Kashanian JA. Recent advances in understanding and managing male infertility. F1000Research. 2019;8. 10.12688/f1000research.17076.1 PMC6524745 31143441

[3] Naz MSG, Ozgoli G, Sayehmiri K (2020). Prevalence of infertility in Iran: a systematic review and meta-analysis. Urol J.

[4] Aliakbari F, Emadeddin M, Azizi F, Amiri RS, Taghizabet N, Hosseini J (2021). A survey on infertility in men and its relation to risk factors in selected provinces of Iran. JBRA Assist Reprod.

[5] Bellver J, Donnez J, Introduction (2019). Infertility etiology and offspring health. Fertil Steril.

[6] Westphal LM, Polan ML, Trant AS, Mooney S (2004). A nutritional supplement for improving fertility in women. J Reprod Med.

[7] Skoracka K, Ratajczak AE, Rychter AM, Dobrowolska A, Krela-Kaźmierczak I (2021). Female fertility and the nutritional approach: the most essential aspects. Adv Nutr.

[8] Bhardwaj JK, Panchal H, Saraf P (2021). Ameliorating effects of natural antioxidant compounds on female infertility: a review. Reproductive Sci.

[9] Bhardwaj JK, Panchal H, Saraf P (2021). Ameliorating effects of natural antioxidant compounds on female infertility: a review. Reproductive Sci.

[10] Ruder EH, Hartman TJ, Reindollar RH, Goldman MB (2014). Female dietary antioxidant intake and time to pregnancy among couples treated for unexplained infertility. Fertil Steril.

[11] Cicek N, Eryilmaz OG, Sarikaya E, Gulerman C, Genc Y (2012). Vitamin E effect on controlled ovarian stimulation of unexplained infertile women. J Assist Reprod Genet.

[12] Peres H, Foss M, Pereira L, Viana C (2017). An update-the role of nutrients crucial in the infertility of couples-New insights for the effects of Iodine, Selenium, Omega 3 fatty acids and Magnesium. J Nutr Health Food Sci.

[13] Elomda AM, Saad MF, Saeed AM, Elsayed A, Abass AO, Safaa HM (2018). Antioxidant and developmental capacity of retinol on the in vitro culture of rabbit embryos. Zygote.

[14] Talebi S, Arab A, Sorraya N (2022). The association between dietary antioxidants and semen parameters: a cross-sectional study among Iranian infertile men. Biol Trace Elem Res.

[15] Ebrahimi Z, Masoodi M, Aslani Z, Naghshi S, Khalighi Sikaroudi M, Shidfar F (2022). Association between dietary antioxidant index and risk of Helicobacter pylori Infection among adults: a case–control study. BMC Gastroenterol.

[16] Vahid F, Rahmani D, Hekmatdoost A (2021). The association between dietary antioxidant index (DAI) and nonalcoholic fatty Liver Disease (NAFLD) onset; new findings from an incident case-control study. Clin Nutr ESPEN.

[17] Rahimlou M, Sohaei S, Nasr-Esfahani M, Nouri M (2019). Dietary antioxidant intake in relation to semen quality parameters in infertile men: a cross-sectional study. Clin Nutr Res.

[18] Nadjarzadeh A, Mehrsai A, Mostafavi E, Gohari MR, Shidfar F (2013). The association between dietary antioxidant intake and semen quality in infertile men. Med J Islamic Repub Iran.

[19] Haeri F, Nouri M, Nezamoleslami S, Moradi A, Ghiasvand R (2022). Role of dietary antioxidants and vitamins intake in semen quality parameters: a cross-sectional study. Clin Nutr ESPEN.

[20] Mirmiran P, Esfahani FH, Mehrabi Y, Hedayati M, Azizi F (2010). Reliability and relative validity of an FFQ for nutrients in the Tehran lipid and glucose study. Public Health Nutr.

[21] Wright ME, Mayne ST, Stolzenberg-Solomon RZ, Li Z, Pietinen P, Taylor PR (2004). Development of a comprehensive dietary antioxidant index and application to Lung cancer risk in a cohort of male smokers. Am J Epidemiol.

[22] Aljaser F, Tabassum H, Fatima S, Abudawood M, Banu N (2021). Effect of trace elements on the seminal oxidative status and correlation to sperm motility in infertile Saudi males. Saudi J Biol Sci.

[23] Iavarone I, Greco PF, La Verde M, Morlando M, Torella M, De Franciscis P (2023). Correlations between Gut Microbial Composition, Pathophysiological and Surgical aspects in Endometriosis: a review of the literature. Medicina.

[24] Hosseini B, Eslamian G. Association of dietary factors with male and female infertility: review of current evidence. Thrita. 2014;3(3).

[25] Henkel R, Sandhu IS, Agarwal A (2019). The excessive use of antioxidant therapy: a possible cause of male infertility?. Andrologia.

[26] Abdollahpour I, Nedjat S, Salimi Y, Mansournia MA, Vahid F, Weinstock-Guttman B (2022). The role of dietary antioxidant index and index of nutritional quality in MS onset: finding from an Iranian population-based incident case–control study. Nutr Neurosci.

[27] Mier-Cabrera J, Aburto-Soto T, Burrola-Méndez S, Jiménez-Zamudio L, Tolentino MC, Casanueva E (2009). Women with endometriosis improved their peripheral antioxidant markers after the application of a high antioxidant diet. Reproductive Biology and Endocrinology.

[28] Grieger JA, Grzeskowiak LE, Wilson RL, Bianco-Miotto T, Leemaqz SY, Jankovic-Karasoulos T (2019). Maternal selenium, copper and zinc concentrations in early pregnancy, and the association with fertility. Nutrients.

[29] Rai T, Khatoon F, Nishad S, Singh A, Sharma B, Gulati S (2023). Role of antioxidants in the treatment of unexplained infertility. J South Asian Federation Obstet Gynecol.

[30] Wang Y, Sharma RK, Falcone T, Goldberg J, Agarwal A (1997). Importance portance of reactive oxygen species in the peritoneal fluid of women with endometriosis or idiopathic infertility. Fertil Steril.

[31] Luck M, Zhao Y (1993). Identification and measurement of collagen in the bovine corpus luteum and its relationship with ascorbic acid and tissue development. Reproduction.

[32] Wang L, Tang J, Wang L, Tan F, Song H, Zhou J (2021). Oxidative stress in oocyte aging and female reproduction. J Cell Physiol.

[33] Symonds DA, Merchenthaler I, Flaws JA (2008). Methoxychlor and estradiol induce oxidative stress DNA damage in the mouse ovarian surface epithelium. Toxicol Sci.

[34] Yeh J, Bowman MJ, Browne RW, Chen N (2005). Reproductive aging results in a reconfigured ovarian antioxidant defense profile in rats. Fertil Steril.

[35] Sahin K, Orhan C, Kucuk O, Tuzcu M, Sahin N, Ozercan IH (2022). Effects of magnesium picolinate, zinc picolinate, and selenomethionine co-supplementation on reproductive hormones, and glucose and lipid metabolism-related protein expressions in male rats fed a high-fat diet. Food Chemistry: Molecular Sciences.

[36] Farsinejad-Marj M, Azadbakht L, Mardanian F, Saneei P, Esmaillzadeh A (2020). Clinical and metabolic responses to magnesium supplementation in women with polycystic ovary syndrome. Biol Trace Elem Res.

